# Vitamin D deficiency in newly diagnosed childhood-onset systemic lupus erythematosus: prevalence and clinical associations

**DOI:** 10.1007/s00431-026-07066-3

**Published:** 2026-05-15

**Authors:** Thawin Ratanaphisit, Maynart Sukharomana, Nuntawan Piyaphanee, Sirikarn Tangcheewinsirikul, Sirirat Charuvanij

**Affiliations:** 1 Department of Pediatrics, Faculty of Medicine Siriraj Hospital, Mahidol University, Bangkok, Thailand; 2 Division of Rheumatology, Department of Pediatrics, Faculty of Medicine Siriraj Hospital, Mahidol University, 2 Wanglang Road, Bangkok Noi, Bangkok, 10700 Thailand; 3 Division of Nephrology, Department of Pediatrics, Faculty of Medicine Siriraj Hospital, Mahidol University, Bangkok, Thailand

**Keywords:** 25-hydroxyvitamin D, Childhood-onset SLE, Lupus nephritis, Systemic lupus erythematosus, Vitamin D

## Abstract

This study aimed to determine the prevalence of vitamin D deficiency and identify its associated factors in patients with newly diagnosed childhood-onset systemic lupus erythematosus (c-SLE). This retrospective study included patients with c-SLE aged ≤ 18 years treated at an academic tertiary center between January 2013 and December 2023. Vitamin D deficiency was defined as a serum 25-hydroxyvitamin D (25-OHD) level < 20 ng/mL. A total of 192 patients with c-SLE were included (87% female; mean [SD] age, 12.1 [3.0] years). Lupus nephritis (LN) was identified in 92 patients (48.2%). The median serum 25-OHD level was 15 ng/mL (IQR, 11.2–22.8), and vitamin D deficiency was identified in 126 patients (65.6%). Compared to those without vitamin D deficiency, deficient patients were older and had higher body mass index, more frequent LN, and higher urine protein-to-creatinine ratio (UPCR; *P* < 0.05). These patients also had lower estimated glomerular filtration rate and serum complement 3 levels (*P* < 0.05). Serum 25-OHD levels were negatively correlated with UPCR (*ρ* =  − 0.38, *P* < 0.001). On multivariable logistic regression, increased UPCR was independently associated with vitamin D deficiency (adjusted odds ratio, 1.54; 95% CI, 1.15–2.24; *P* = 0.011). Patients with moderate (SLEDAI-2 K, 7–12) and high disease activity (SLEDAI-2 K ≥ 13) had a higher prevalence of vitamin D deficiency (*P* = 0.002 and *P* < 0.001, respectively).

*Conclusion*: Vitamin D deficiency was prevalent in newly diagnosed c-SLE and was associated with LN, proteinuria, and moderate to high disease activity.

**Table:** 

**What is Known:** • *Childhood-onset systemic lupus erythematosus (c-SLE) is a complex autoimmune connective tissue disease in which vitamin D has been implicated in immune dysregulation*.	
**What is New:** • *Vitamin D deficiency was identified in 65.6% of newly diagnosed Southeast Asian c-SLE*. • *c-SLE patients with LN had a higher prevalence of vitamin D deficiency than those without LN, particularly those with proteinuria*.

**Supplementary Information:**

The online version contains supplementary material available at 10.1007/s00431-026-07066-3.

## Introduction

Childhood-onset systemic lupus erythematosus (c-SLE) is an autoimmune inflammatory connective tissue disease defined by onset before 18 years of age. c-SLE accounts for up to 20% of all SLE cases and occurs more commonly in females than males [1]. It is generally characterized by a higher prevalence of renal, hematologic, and neurological involvement and greater organ damage than adult-onset disease [2–4]. c-SLE has been reported as the leading cause of morbidity among children and adolescents who have autoimmune rheumatic diseases and require intensive care admission [5]. The pathogenesis of c-SLE is complex, involving monogenic and polygenic predisposition as well as increased type I interferon signaling [6]. Immune dysregulation contributes to autoantibody production and complement-activated immune complex deposition, resulting in organ inflammation and damage.

Vitamin D regulates calcium and skeletal homeostasis and serves as an immunomodulator that affects both innate and adaptive immunity [7]. Several immune cells, including dendritic cells, B lymphocytes, and T lymphocytes, express vitamin D receptors, which supports the immunomodulatory role of vitamin D [8]. Vitamin D may be involved in the pathogenesis of SLE through multiple mechanisms. These include reduction of B cell proliferation and autoantibody production, enhancement of regulatory T cells, increased interleukin-10 secretion, and suppression of T-helper 1 and T-helper 17 responses [9].

Patients with c-SLE are generally at risk of vitamin D deficiency because of several factors, such as avoidance of sun exposure, limited outdoor physical activity, and corticosteroid treatment. Studies have found that vitamin D status is associated with SLE disease activity [10–13]. Patients with c-SLE experiencing disease flares have lower 25-hydroxyvitamin D (25-OHD) levels than those with inactive disease [11]. Furthermore, vitamin D supplementation has been shown to improve disease activity and lessen fatigue in patients with c-SLE [14].

Although previous studies have reported a high prevalence of low serum vitamin D levels in c-SLE, vitamin D measurements in most reports were obtained after treatment initiation or concurrently with vitamin D supplementation [11–13]. Consequently, treatment-related factors such as corticosteroid exposure, immunosuppressive therapy, and concurrent vitamin D supplementation may have substantially influenced circulating vitamin D concentrations. This methodological limitation prevents accurate assessment of the true prevalence of vitamin D deficiency at diagnosis and complicates interpretation of the relationship between vitamin D status and disease characteristics. Moreover, data on vitamin D status in Southeast Asian patients with c-SLE who live near the equator with year-round sun exposure are limited. Therefore, this study aimed to evaluate vitamin D status and explore factors associated with vitamin D deficiency in patients with newly diagnosed c-SLE.

## Methods

### Study design and setting

This retrospective study was conducted at the Department of Pediatrics, Faculty of Medicine Siriraj Hospital, Mahidol University, the largest super-tertiary academic center in Bangkok, Thailand. The research protocol was approved by the Siriraj Institutional Review Board (Si 900/2024) and was performed in accordance with the Declaration of Helsinki. The requirement for informed consent and assent was waived because of the retrospective design. Data were collected from January 2013 to December 2023 from electronic medical records. Cases were identified using the International Statistical Classification of Diseases, Tenth Revision, codes M32.1 (SLE with organ or system involvement), M32.8 (other forms of SLE), and M32.9 (unspecified SLE).

### Participants

Patients with c-SLE diagnosed before 18 years of age were included. SLE was classified according to the 1997 American College of Rheumatology [15], the 2012 Systemic Lupus International Collaborating Clinics [16], or the 2019 European League against Rheumatism/American College of Rheumatology criteria [17]. Patients were excluded if they had malabsorption syndrome, hyperparathyroidism, or chronic kidney disease (GFR < 60 mL/min/1.73 m2 for > 3 months); were receiving vitamin D supplementation; or did not have a serum 25-OHD level obtained at the time of diagnosis.

### Data collection

Demographic data, clinical characteristics, and laboratory findings at the time of SLE diagnosis were collected. Demographic data included sex, age at diagnosis, weight, height, and underlying diseases. Clinical characteristics included fever, mucocutaneous, hematologic, renal, musculoskeletal, chest, cardiovascular, neuropsychiatric, and gastrointestinal involvement. Lupus nephritis (LN) was classified according to the International Society of Nephrology/Renal Pathology Society classification into six classes: Class I (minimal mesangial), Class II (mesangial proliferative), Class III (focal), Class IV (diffuse), Class V (membranous), and Class VI (advanced sclerosing) [18]. Estimated glomerular filtration rates were calculated using the modified Schwartz formula [19].

Laboratory results at initial diagnosis were recorded, including complete blood count, erythrocyte sedimentation rate, C-reactive protein, urinalysis, urine protein, urine creatinine, blood urea nitrogen, serum creatinine, antinuclear antibody, anti-double-stranded DNA, and complement levels. Disease activity was evaluated using the Systemic Lupus Erythematosus Disease Activity Index 2000 (SLEDAI-2 K) [20]. Disease severity was categorized as mild (SLEDAI-2 K ≤ 6), moderate (SLEDAI-2 K 7–12), or high (SLEDAI-2 K ≥ 13) [21].

### Vitamin D assessment and classification

Serum 25-OHD was measured by immunoassay (Elecsys; Roche Diagnostics, Basel, Switzerland). The mean intra-assay and inter-assay coefficients of variation were 1.1–8.9% and 2.2–10.8%, respectively. Vitamin D deficiency and insufficiency were defined as serum 25-OHD levels of < 20 ng/mL and 20 to < 30 ng/mL, respectively [22]. A normal vitamin D level was defined as a serum 25-OHD level ≥ 30 ng/mL [22].

### Statistical analysis

The sample size was calculated on the basis of an estimated prevalence of vitamin D deficiency in SLE of 38.6% [23]. The allowable difference between the estimated and true prevalence was set at no more than 15%, with a precision of 0.058 and a significance level of 0.05. The calculated sample size was 272.

Statistical analyses were performed using IBM SPSS Statistics version 20 (IBM Corp, Armonk, NY, USA) and RStudio. Categorical data were reported as frequencies with percentages. Continuous data were presented as mean (SD) for normally distributed data or median (IQR) for nonnormally distributed data. Continuous variables were compared between groups using the independent samples t test or Mann–Whitney *U* test, and categorical variables were compared using the chi-square test. Pearson correlation coefficient (r) or Spearman correlation coefficient (*ρ*) was used, as appropriate, to evaluate correlations between variables. Values of 0.6–1.0 indicated a strong correlation, 0.4–0.59 a moderate correlation, 0.2–0.39 a weak correlation, and < 0.2 no correlation. Univariable logistic regression analysis was performed prior to the multivariable logistic regression analysis. Variables for the univariable logistic regression analysis were pre-specified based on previous studies and clinical relevance. Variables with *P* values less than 0.05 were selected for the multivariable logistic regression analysis. Multivariable logistic regression analysis using the enter method was performed to explore factors associated with vitamin D deficiency. The model was adjusted for age, sex, body mass index, mucocutaneous involvement, urine protein-to-creatinine ratio (UPCR), and serum complement 3 (C3). A *P* value < 0.05 was considered statistically significant.

## Results

### Demographic and clinical characteristics

Of 267 patients with c-SLE, 71 did not have a 25-OHD level obtained at the time of SLE diagnosis and 4 were receiving vitamin D supplementation (Supplemental Table 1 and Supplemental Fig. 1). Therefore, 192 patients with c-SLE were included in this study. Most patients were female (*n* = 167, 87%), and the mean (SD) age was 12.1 (3.0) years. The most common clinical manifestations were hematologic involvement (156 patients, 81.1%), followed by mucocutaneous involvement (140 patients, 72.9%). Biopsy-proven LN was identified in 92 patients (48.2%), and LN class IV was the most common subtype (57 patients, 29.8%). All patients tested positive for antinuclear antibody, and anti-double-stranded DNA antibodies were detected in 154 patients (80.2%). The median SLEDAI-2 K score was 13 (IQR, 8–17). Demographic and clinical characteristics are presented in Table 1.

**Table 1:** Demographic characteristics, clinical features, and laboratory findings of 192 patients with newly diagnosed childhood-onset systemic lupus erythematosus, stratified by serum 25-hydroxyvitamin D level

Characteristic	Total (*N* = 192)	Vitamin D deficient (25-OHD < 20 ng/mL) (*n* = 126, 65.6%)	Vitamin D nondeficient (25-OHD ≥ 20 ng/mL) (*n* = 66, 34.4%)	*P* value
Demographic characteristics				
Age at diagnosis, y (SD)^a^	12.1 (3)	12.7 (2.7)	11.1 (3.2)	** < 0.001**
Female sex	167 (87)	114 (90.5)	53 (80.3)	**0.047**
BMI, kg/m^2^	18.3 (16–21.7)	19.2 (16.7–22.6)	17.1 (14.9–20.1)	** < 0.001**
Clinical characteristics				
Fever	122 (63.5)	82 (65.6)	40 (60.6)	0.541
Mucocutaneous	140 (72.9)	82 (65.1)	58 (87.9)	** < 0.001**
Oral ulcer	80 (41.9)	40 (32)	40 (60.6)	** < 0.001**
Malar rash	84 (44.2)	43 (34.7)	41 (62.1)	** < 0.001**
Discoid lupus	44 (23)	23 (18.4)	21 (31.8)	**0.036**
Photosensitivity	31 (16.3)	14 (11.3)	17 (25.8)	**0.01**
Non-scarring alopecia	44 (23)	27 (21.6)	14 (25.8)	0.51
Hematologic	156 (81.2)	101 (80.2)	55 (83.3)	0.592
AIHA	70 (36.6)	46 (36.5)	24 (36.9)	0.955
Thrombocytopenia	34 (17.8)	29 (23)	5 (7.7)	**0.015**
Neuropsychiatric	17 (8.9)	12 (9.5)	5 (7.6)	0.652
Musculoskeletal	46 (24)	26 (20.6)	20 (30.3)	0.136
Pulmonary	24 (12.5)	21 (16.7)	3 (4.5)	**0.016**
Cardiac	28 (14.6)	25 (19.8)	3 (4.5)	**0.004**
Gastrointestinal	28 (14.6)	23 (18.3)	5 (7.6)	**0.046**
Renal involvement				
LN	92 (48.2)	70 (56.0)	22 (33.3)	**0.005**
I	1 (0.5)	1 (0.8)	0 (0)	1.000
II	9 (4.7)	5 (4.0)	4 (6.1)	0.779
III, III + V	21 (11)	11 (8.8)	10 (15.2)	0.275
IV, IV + V	57 (29.8)	49 (39.2)	8 (12.1)	** < 0.001**
V	6 (3.1)	5 (4.0)	1 (1.5)	0.617
Proliferative LN^b^	77 (40.3)	60 (48)	17 (25.8)	**0.005**
Laboratory findings				
Hemoglobin, g/dL	9.8 (8.5–11)	9.6 (8.2–11)	10.2 (8.8–11.4)	**0.039**
WBC, × 10^3^/μL	5075 (3295–7742.5)	4815 (3290–7590)	5380 (3315–8400)	0.552
Platelet count, × 10^3^/μL	239 500 (146 000–328 750)	219 500 (132 500–328 000)	260 500 (195 000–328 000)	0.225
ESR, mm/h	60.5 (33–87)	59 (31–87)	62 (35–88)	0.703
Creatinine, mg/dL	0.5 (0.4–0.8)	0.6 (0.5–0.9)	0.5 (0.4–0.6)	** < 0.001**
UPCR, mg/mg	1.1 (0.5–4.2)	1.8 (0.6–4.7)	0.6 (0.2–1)	** < 0.001**
eGFR, mL/min/1.73 m^2^	110 (82–132.7)	104.1 (67.9–129.8)	122.7 (104.5–145.3)	** < 0.001**
C3, mg/dL	36.3 (24.1–53.4)	34.9 (22.5–50)	40.7 (28.6–65.2)	**0.031**
C4, mg/dL	5.5 (3.4–8.3)	5.3 (3.4–8.2)	5.9 (4–8.3)	0.437
Anti-dsDNA antibody positivity	154 (80.2)	106 (84.1)	48 (72.7)	0.091
Disease activity at diagnosis				
SLEDAI-2 K	13 (8–17)	13 (10–17)	12 (6.3–15.8)	0.144

Data are presented as *n* (%) for categorical variables, mean (SD) for normally distributed continuous variables^a^, or median (IQR) for nonnormally distributed continuous variables. Comparisons between the vitamin D-deficient and nondeficient groups were performed using the independent samples *t* test or Mann–Whitney *U* test for continuous variables and the chi-square test for categorical variables. Bold *P* values indicate statistical significance (*P* < 0.05). ^a^Age at diagnosis is the only variable reported as mean (SD); all other continuous variables are reported as median (IQR). ^b^Proliferative LN includes ISN/RPS classes III, III + V, IV, and IV + V. *25-OHD* 25-hydroxyvitamin D, *AIHA* autoimmune hemolytic anemia, *anti-dsDNA* anti-double-stranded DNA, *BMI*, body mass index, *C3* complement 3, *C4* complement 4, *c-SLE* childhood-onset systemic lupus erythematosus, *eGFR* estimated glomerular filtration rate, *ESR* erythrocyte sedimentation rate, *IQR* interquartile range, *LN* lupus nephritis, *SD* standard deviation, *SLEDAI-2K* Systemic Lupus Erythematosus Disease Activity Index 2000, *UPCR* urine protein-to-creatinine ratio, *WBC* white blood cell count

**Fig. 1:**
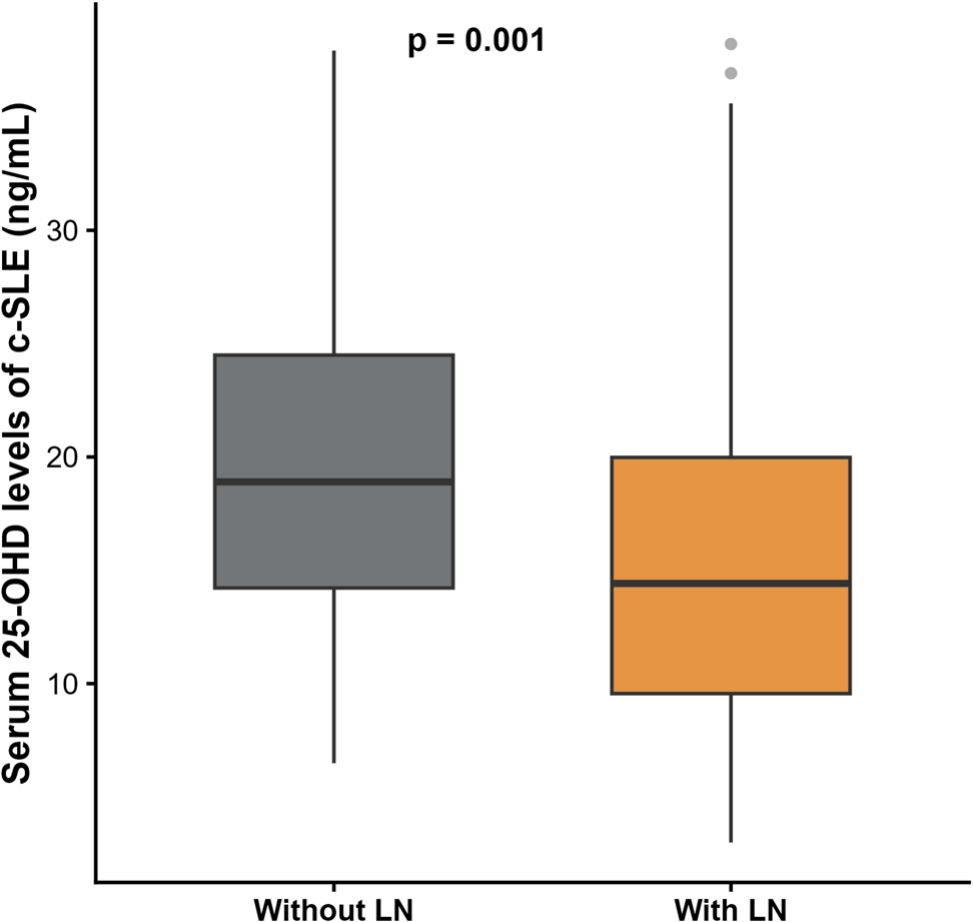
Serum 25-hydroxyvitamin D levels in patients with newly diagnosed childhood-onset systemic lupus erythematosus, stratified by the presence or absence of lupus nephritis. Box plots show the median (horizontal line), interquartile range (box), and range (whiskers) of serum 25-OHD levels (ng/mL). Outliers are shown as individual points. Patients with LN (*n* = 92) had lower median serum 25-OHD levels than those without LN (*n* = 100; *P* = 0.001, Mann–Whitney *U* test). Abbreviations: 25-OHD, 25-hydroxyvitamin D; c-SLE, childhood-onset systemic lupus erythematosus; LN, lupus nephritis

### Vitamin D status

**Fig. 2:**
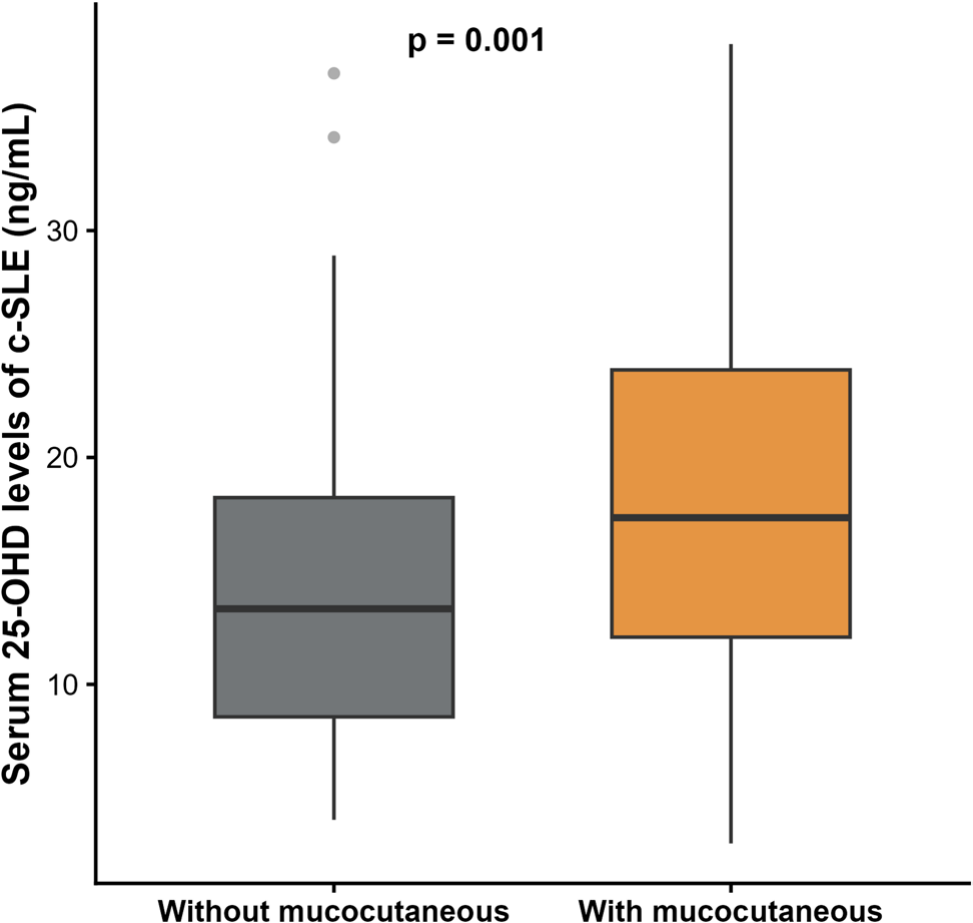
Serum 25-hydroxyvitamin D levels in patients with newly diagnosed childhood-onset systemic lupus erythematosus, stratified by the presence or absence of mucocutaneous involvement. Box plots show the median (horizontal line), interquartile range (box), and range (whiskers) of serum 25-OHD levels (ng/mL). Outliers are shown as individual points. Patients with mucocutaneous involvement (*n* = 140) had higher median serum 25-OHD levels than those without mucocutaneous involvement (*n* = 52; *P* = 0.001, Mann–Whitney *U* test). Abbreviations: 25-OHD, 25-hydroxyvitamin D; c-SLE, childhood-onset systemic lupus erythematosus

The median serum 25-OHD level was 15 ng/mL (IQR, 11.2–22.8), and vitamin D deficiency was identified in 126 patients (65.6%). Vitamin D insufficiency and normal vitamin D were found in 47 (24.5%) patients and 19 patients (9.9%), respectively. The median serum 25-OHD levels measured during the summer in 63 patients were 16 (12.2–21.3) ng/mL. During the rainy season (84 patients) and the winter season (45 patients), the median serum 25-OHD levels were 17.2 (11.3–23.3) ng/mL and 15 (11.2–21.6) ng/mL, respectively. These differences were not statistically significant (*P* = 0.508). Compared with patients without vitamin D deficiency, those with deficiency were older, had higher body mass index, more frequent LN, lower estimated glomerular filtration rates, higher UPCR, and lower C3 levels (*P* < 0.05). A higher prevalence of vitamin D deficiency was observed among patients with LN class IV and IV + V (*P* < 0.001). The distribution of vitamin D status in patients with and without LN is presented in Fig. 1. In contrast, patients without vitamin D deficiency had more frequent mucocutaneous involvement than those with deficiency (*P* < 0.001; Fig. 2). Clinical characteristics of patients with and without vitamin D deficiency are further detailed in Table 1.

### Factors associated with vitamin D deficiency

On multivariable logistic regression analysis (Table 2), UPCR (adjusted odds ratio [aOR], 1.54; 95% CI, 1.15–2.24; *P* = 0.011), age (aOR, 1.45; 95% CI, 1.20–1.81; *P* < 0.001), and serum C3 (aOR, 0.97; 95% CI, 0.95–0.99; *P* = 0.028) were independently associated with vitamin D deficiency in newly diagnosed c-SLE.

**Table 2:** Univariable and multivariable logistic regression analyses of factors associated with vitamin D deficiency (serum 25-hydroxyvitamin D < 20 ng/mL) in 126 patients with newly diagnosed childhood-onset systemic lupus erythematosus

Variable	Univariable logistic regression analysis		Multivariable logistic regression analysis	
	OR (95% CI)	*P* value	aOR (95% CI)	*P* value
Age at diagnosis, y	1.21 (1.08–1.35)	** < 0.001**	1.45 (1.20–1.81)	** < 0.001**
Female sex	2.33 (1.00–5.52)	0.051	—	—
BMI, kg/m^2^	1.12 (1.04–1.22)	**0.004**	1.14 (1.01–1.32)	0.054
Mucocutaneous involvement	0.26 (0.11–0.59)	** < 0.001**	0.18 (0.04–0.65)	**0.015**
Lupus nephritis	2.55 (1.37–4.74)	**0.003**	—	—
UPCR, mg/mg	1.70 (1.25–2.30)	** < 0.001**	1.54 (1.15–2.24)	**0.011**
Serum C3, mg/dL	0.99 (0.97–1.00)	0.06	0.97 (0.95–0.99)	**0.028**

The multivariable model was adjusted for all variables listed in the table. Bold *P* values indicate statistical significance (*P* < 0.05). The em dash (—) indicates that the variable was not included in the multivariable model. *25-OHD* 25-hydroxyvitamin D, *aOR* adjusted odds ratio, *BMI* body mass index, *C3* complement 3, *CI* confidence interval, *OR* odds ratio, *UPCR* urine protein-to-creatinine ratio

**Fig. 3:**
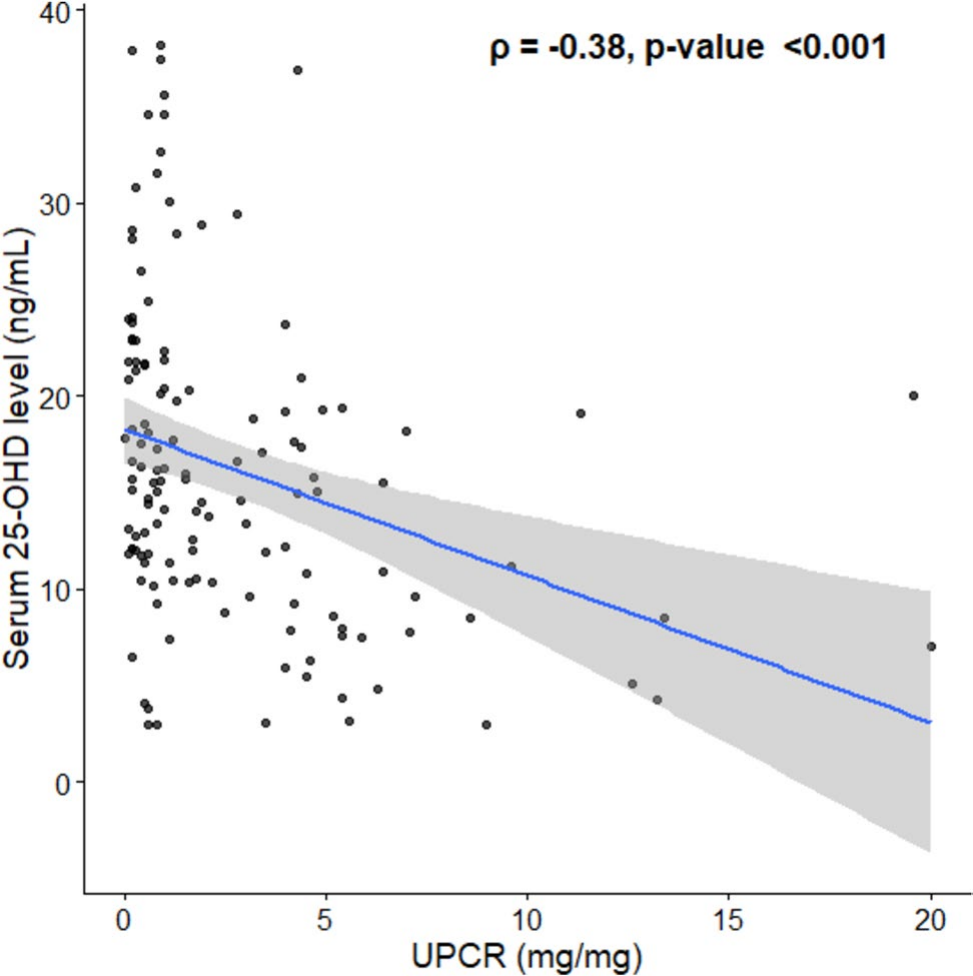
Correlation between serum 25-hydroxyvitamin D levels and urine protein-to-creatinine ratio in 192 patients with newly diagnosed childhood-onset systemic lupus erythematosus. Each point represents an individual patient. The blue line represents the regression line, and the shaded area indicates the 95% confidence interval. Serum 25-OHD levels were negatively correlated with UPCR (Spearman *ρ* =  − 0.38, *P* < 0.001). Abbreviations: 25-OHD, 25-hydroxyvitamin D; c-SLE, childhood-onset systemic lupus erythematosus; UPCR, urine protein-to-creatinine ratio

**Fig. 4:**
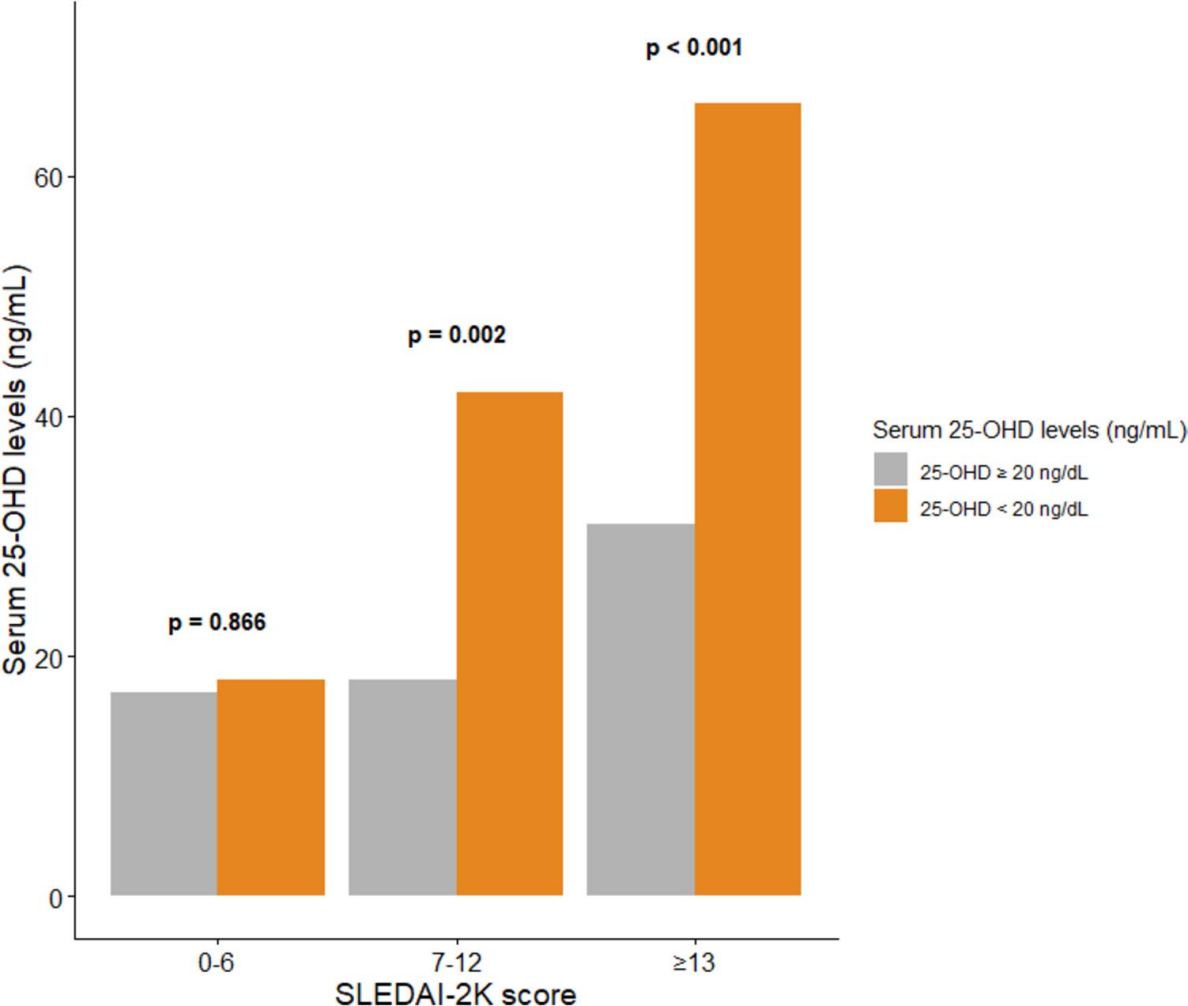
Distribution of vitamin D status by disease activity category in 192 patients with newly diagnosed childhood-onset systemic lupus erythematosus. Bars represent the number of patients with vitamin D deficiency (serum 25-OHD < 20 ng/mL; orange) and vitamin D nondeficiency (serum 25-OHD ≥ 20 ng/mL; gray) within each disease activity category defined by the SLEDAI-2 K score: mild (0–6), moderate (7–12), and high (≥ 13). The prevalence of vitamin D deficiency was higher among patients with moderate (*P* = 0.002) and high disease activity (*P* < 0.001) compared with those with mild disease activity (*P* = 0.866). Abbreviations: 25-OHD, 25-hydroxyvitamin D; c-SLE, childhood-onset systemic lupus erythematosus; SLEDAI-2 K, Systemic Lupus Erythematosus Disease Activity Index 2000

Serum 25-OHD levels were negatively correlated with UPCR (*ρ* =  − 0.38, *P* < 0.001; Fig. 3) and weakly positively correlated with C3 (*ρ* = 0.20, *P* = 0.009). No correlation was found between serum 25-OHD levels and SLEDAI-2 K scores (*ρ* =  − 0.11, *P* = 0.11). In subgroup analysis, patients with moderate and high disease activity had a higher prevalence of vitamin D deficiency (*P* = 0.002 and *P* < 0.001, respectively; Fig. 4).

## Discussion

Our study found that vitamin D deficiency was highly prevalent in newly diagnosed Southeast Asian patients with c-SLE, affecting 65.6% of the cohort. Patients with LN had a higher prevalence of vitamin D deficiency than those without LN, particularly those with proteinuria. Higher UPCR and lower C3 levels were correlated with lower 25-OHD levels. Vitamin D deficiency was also more prevalent among patients with moderate to high disease activity.

The prevalence of vitamin D deficiency varies across studies. Global data from Cui et al. indicated that the prevalence of vitamin D deficiency among children and adolescents was 48.5% (95% CI, 42.5–54.5%) [24]. Because Thailand is located near the equator with year-round sunlight exposure, the prevalence of vitamin D deficiency in healthy Thai children has been reported as only 19.5% [25]. However, our study found a prevalence of 65.6% in patients with c-SLE, which is considerably higher than that in the healthy Thai population. This finding supports the potential role of vitamin D in immune dysregulation and the pathogenesis of SLE.

The reported prevalence of vitamin D deficiency in c-SLE varies across studies, possibly because of geographic and seasonal differences as well as variations in study design, timing of serum 25-OHD measurement, and the definition of vitamin D deficiency used. Hamza et al. studied 60 Egyptian patients with c-SLE and reported a prevalence of 73.3% for serum 25-OHD < 30 ng/mL [26]. Robinson et al. reported a prevalence of 30% (25-OHD < 20 ng/mL) among 201 patients with c-SLE from North American centers [27]. Consistent with our findings, Jiang et al. reported that 61.3% of patients with c-SLE had vitamin D deficiency based on a serum 25-OHD level < 20 ng/mL [10].

Vitamin D deficiency in c-SLE is attributed to several factors, including avoidance of sunlight exposure, corticosteroid treatment, and disease severity. Tabra et al. reported that patients with c-SLE who had active disease had lower vitamin D levels than those with inactive disease [11]. Lin et al. found that 25-OHD levels were negatively correlated with SLE disease activity in both active and inactive disease among 35 patients with c-SLE [12]. In a small study of 28 patients with c-SLE, 25-OHD levels correlated inversely with disease activity, and most patients showed favorable disease activity after 3 months of vitamin D3 treatment [13]. Furthermore, vitamin D supplementation has been shown to reduce disease activity and improve fatigue in patients with c-SLE [14]. Although serum 25-OHD levels were not correlated with SLEDAI-2 K scores as a continuous variable, vitamin D deficiency was more prevalent among c-SLE patients with moderate to high disease activity. This may reflect a non-linear or threshold relationship rather than a direct linear association. These findings suggest that vitamin D deficiency may be associated with more active disease; however, causality cannot be established, and the results should be interpreted with caution. A higher proportion of females was observed in the vitamin D deficiency group compared to the non-deficiency group. However, this association was not statistically significant in the univariable logistic regression analysis and was therefore not included in the multivariable model. This suggests that the observed difference may be due to chance or confounded by other factors.

Our study found that patients with c-SLE and LN were more likely to have vitamin D deficiency, and that higher UPCR and lower C3 levels were correlated with lower 25-OHD levels. Proliferative LN was associated with vitamin D deficiency, consistent with previous findings [28]. Reduced vitamin D levels have been identified as a risk factor for LN in c-SLE, with the lowest 25-OHD levels observed in LN class V [10]. During active disease, patients with c-SLE and LN had lower 25-OHD levels than those without LN [12]. A negative correlation between 24-h urine protein and 25-OHD has been reported in c-SLE; however, that study was conducted in patients with established c-SLE receiving various dosages of corticosteroids [11].

Urinary loss of vitamin D-binding protein may explain the observed vitamin D deficiency in patients with c-SLE and LN [29]. Robinson et al. further demonstrated a negative correlation between 25-OHD levels and the urinary vitamin D-binding protein-to-creatinine ratio in c-SLE, indicating loss of protein-bound vitamin D metabolites [28]. In renal histopathology, downregulation of vitamin D receptor expression has been found in patients with LN and was negatively correlated with severity [30]. Vitamin D has also been shown to play a protective role against podocyte injury induced by autoantibodies by decreasing aberrant autophagy in LN [31]. Taken together, these findings provide important insights into the role of vitamin D signaling in the pathogenesis and progression of LN.

This study has several limitations. Because of the retrospective cohort design, a healthy control group was not included for direct comparison with patients with c-SLE. This study was conducted at a single tertiary referral center with a relatively high proportion of c-SLE patients with LN, which may reflect more severe disease. Therefore, referral bias cannot be excluded and the results may have limited generalizability. The exact timing of the initiation of corticosteroid or immunosuppressive therapy was not fully recorded. Therefore, we cannot exclude the possibility that some patients received corticosteroid treatment prior to referral. Although the calculated sample size was not fully achieved, the study included a relatively large cohort of newly diagnosis of c-SLE patients. The reduced sample size may have limited statistical power to detect modest associations in regression analyses. A substantial proportion of eligible patients were excluded due to missing baseline 25-OHD levels. Differences in baseline characteristics between included and excluded patients suggest potential selection bias, which may have affected both prevalence estimates and identified associations. Therefore, the findings should be interpreted with caution. Several potential confounding factors influencing vitamin D status, including sunlight exposure, dietary intake, and possible corticosteroid use prior to referral were not available and could not be accounted for in this study. Although Thailand is located near the equator with year-round sun exposure, seasonal and behavioral factors such as sun protection and dietary intake may still affect vitamin D levels.

Despite these limitations, our study has notable strengths. It specifically focused on newly diagnosed c-SLE, an area in which published data remain scarce. The relationship between clinical characteristics and disease severity at diagnosis and serum 25-OHD levels was demonstrated in a Southeast Asian c-SLE population. Furthermore, because Thailand is located near the equator, seasonal variation is expected to have had minimal impact on serum 25-OHD measurements in this cohort.

In summary, vitamin D deficiency was prevalent in newly diagnosed c-SLE, particularly among patients with LN, proteinuria, and moderate to high disease activity. These findings support the consideration of screening for vitamin D deficiency at diagnosis in patients with c-SLE. Longitudinal studies are needed to explore the trajectory of serum 25-OHD levels and disease activity in c-SLE. The impact of vitamin D deficiency on bone health in this population also warrants further investigation.

## Supplementary Information

Below is the link to the electronic supplementary material.

Supplementary file1 (DOCX 35 KB)

## Data Availability

The datasets used and analyzed during the current study are available from the corresponding author on reasonable request.

## Abbreviations

c-SLE: Childhood-onset systemic lupus erythematosus
C3: Complement 3
LN: Lupus nephritis
SLE: Systemic lupus erythematosus
SLEDAI-2K: Systemic Lupus Erythematosus Disease Activity Index 2000
25-OHD: 25-hydroxyvitamin D
UPCR: Urine protein-to-creatinine ratio

## References

[1] Barber MRW, Drenkard C, Falasinnu T, Hoi A, Mak A, Kow NY et al (2021) Global epidemiology of systemic lupus erythematosus. Nat Rev Rheumatol 17(9):515–532. 10.1038/s41584-021-00668-1 34345022 10.1038/s41584-021-00668-1 PMC8982275

[2] Na Nakorn K, Piyaphanee N, Sukharomana M, Pinpatanapong R, Charuvanij S (2023) Outcomes of achieving lupus low disease activity state and damage accrual in childhood-onset systemic lupus erythematosus. Clin Rheumatol 42(6):1655–1664. 10.1007/s10067-023-06533-8 36780064 10.1007/s10067-023-06533-8

[3] Sukharomana M, Vonginyoo S, Piyaphanee N, Charuvanij S (2024) Musculoskeletal manifestations in childhood-onset systemic lupus erythematosus: an in-depth exploration. Ital J Pediatr 50(1):149. 10.1186/s13052-024-01725-7 39152510 10.1186/s13052-024-01725-7 PMC11330147

[4] Tang SP, Lim SC, Arkachaisri T (2021) Childhood-onset systemic lupus erythematosus: Southeast Asian perspectives. J Clin Med 10(4):559. 10.3390/jcm10040559 33546120 10.3390/jcm10040559 PMC7913223

[5] Buranapattama T, Phumeetham S, Piyaphanee N, Sukharomana M, Charuvanij S (2025) Mortality in children and adolescents with autoimmune inflammatory rheumatic diseases admitted to the pediatric intensive care unit. Pediatr Rheumatol Online J 23(1):20. 10.1186/s12969-025-01068-5 39979968 10.1186/s12969-025-01068-5 PMC11843957

[6] Robinson GA, Knight A, Tucker LB, Belot A, Isenberg DA, Ciurtin C (2026) Insights into the pathogenesis of childhood-onset SLE in the past decade. Nat Rev Rheumatol 22(1):26–41. 10.1038/s41584-025-01321-x 41258446 10.1038/s41584-025-01321-x

[7] El Kababi S, El Ouali EM, Kartibou J, Lamiri A, Deblij S, Supriya R et al (2025) A systematic review and meta-analysis of the effects of vitamin D on systemic lupus erythematosus. Nutrients. 10.3390/nu17172794 40944182 10.3390/nu17172794 PMC12430488

[8] Ghaseminejad-Raeini A, Ghaderi A, Sharafi A, Nematollahi-Sani B, Moossavi M, Derakhshani A et al (2023) Immunomodulatory actions of vitamin D in various immune-related disorders: a comprehensive review. Front Immunol 14:950465. 10.3389/fimmu.2023.950465 37520529 10.3389/fimmu.2023.950465 PMC10379649

[9] Lupu VV, Lupu A, Jechel E, Starcea IM, Stoleriu G, Ioniuc I et al (2024) The role of vitamin D in pediatric systemic lupus erythematosus - a double pawn in the immune and microbial balance. Front Immunol 15:1373904. 10.3389/fimmu.2024.1373904 38715605 10.3389/fimmu.2024.1373904 PMC11074404

[10] Jiang L, Zhi S, Wei C, Rong Z, Zhang H (2023) Serum 25(OH)D levels are associated with disease activity and renal involvement in initial-onset childhood systemic lupus erythematosus. Front Pediatr 11:1252594. 10.3389/fped.2023.1252594 38111622 10.3389/fped.2023.1252594 PMC10725985

[11] Tabra SAA, Abdelnabi HH, Darwish NFM, El-Barbary AM, AbdelGhafar MT, Abu-Zaid MH (2020) Juvenile lupus and serum vitamin D levels: a cross-sectional study. Lupus 29(13):1752–1758. 10.1177/0961203320957721 32924829 10.1177/0961203320957721

[12] Lin TC, Wu JY, Kuo ML, Ou LS, Yeh KW, Huang JL (2018) Correlation between disease activity of pediatric-onset systemic lupus erythematosus and level of vitamin D in Taiwan: a case-cohort study. J Microbiol Immunol Infect 51(1):110–114. 10.1016/j.jmii.2015.12.005 27147283 10.1016/j.jmii.2015.12.005

[13] AlSaleem A, AlE’ed A, AlSaghier A, Al-Mayouf SM (2015) Vitamin D status in children with systemic lupus erythematosus and its association with clinical and laboratory parameters. Clin Rheumatol 34(1):81–84. 10.1007/s10067-014-2811-z 25367346 10.1007/s10067-014-2811-z

[14] Lima GL, Paupitz J, Aikawa NE, Takayama L, Bonfa E, Pereira RM (2016) Vitamin D supplementation in adolescents and young adults with juvenile systemic lupus erythematosus for improvement in disease activity and fatigue scores: a randomized, double-blind, placebo-controlled trial. Arthritis Care Res (Hoboken) 68(1):91–98. 10.1002/acr.22621 25988278 10.1002/acr.22621

[15] Hochberg MC (1997) Updating the American College of Rheumatology revised criteria for the classification of systemic lupus erythematosus. Arthritis Rheum 40(9):1725. 10.1002/art.1780400928 9324032 10.1002/art.1780400928

[16] Petri M, Purvey S, Fang H, Magder LS (2012) Predictors of organ damage in systemic lupus erythematosus: the Hopkins Lupus Cohort. Arthritis Rheum 64(12):4021–4028. 10.1002/art.34672 22932985 10.1002/art.34672 PMC3510359

[17] Aringer M, Costenbader K, Daikh D, Brinks R, Mosca M, Ramsey-Goldman R et al (2019) 2019 European League Against Rheumatism/American College of Rheumatology classification criteria for systemic lupus erythematosus. Ann Rheum Dis 78(9):1151–1159. 10.1136/annrheumdis-2018-214819 31383717 10.1136/annrheumdis-2018-214819

[18] Weening JJ, D’Agati VD, Schwartz MM, Seshan SV, Alpers CE, Appel GB et al (2004) The classification of glomerulonephritis in systemic lupus erythematosus revisited. Kidney Int 65(2):521–530. 10.1111/j.1523-1755.2004.00443.x 14717922 10.1111/j.1523-1755.2004.00443.x

[19] Schwartz GJ, Work DF (2009) Measurement and estimation of GFR in children and adolescents. Clin J Am Soc Nephrol 4(11):1832–1843. 10.2215/cjn.01640309 19820136 10.2215/CJN.01640309

[20] Gladman DD, Ibañez D, Urowitz MB (2002) Systemic lupus erythematosus disease activity index 2000. J Rheumatol 29(2):288–291 11838846

[21] Fanouriakis A, Kostopoulou M, Alunno A, Aringer M, Bajema I, Boletis JN et al (2019) 2019 update of the EULAR recommendations for the management of systemic lupus erythematosus. Ann Rheum Dis 78(6):736–745. 10.1136/annrheumdis-2019-215089 30926722 10.1136/annrheumdis-2019-215089

[22] Holick MF (2009) Vitamin D status: measurement, interpretation, and clinical application. Ann Epidemiol 19(2):73–78. 10.1016/j.annepidem.2007.12.001 18329892 10.1016/j.annepidem.2007.12.001 PMC2665033

[23] Shahin D, El-Farahaty RM, Houssen ME, Machaly SA, Sallam M, ElSaid TO et al (2017) Serum 25-OH Vitamin D level in treatment-naïve systemic lupus erythematosus patients: relation to disease activity, IL-23 and IL-17. Lupus 26(9):917–926. 10.1177/0961203316682095 27927883 10.1177/0961203316682095

[24] Cui A, Zhang T, Xiao P, Fan Z, Wang H, Zhuang Y (2023) Global and regional prevalence of vitamin D deficiency in population-based studies from 2000 to 2022: a pooled analysis of 7.9 million participants. Front Nutr 10:1070808. 10.3389/fnut.2023.1070808 37006940 10.3389/fnut.2023.1070808 PMC10064807

[25] Reesukumal K, Manonukul K, Jirapongsananuruk O, Krobtrakulchai W, Hanyongyuth S, Chatsiricharoenkul S et al (2015) Hypovitaminosis D in healthy children in Central Thailand: prevalence and risk factors. BMC Public Health 15:248. 10.1186/s12889-015-1588-6 25886311 10.1186/s12889-015-1588-6 PMC4364485

[26] Hamza RT, Awwad KS, Ali MK, Hamed AI (2011) Reduced serum concentrations of 25-hydroxy vitamin D in Egyptian patients with systemic lupus erythematosus: relation to disease activity. Med Sci Monit 17(12):Cr711-718. 10.12659/msm.882131 22129903 10.12659/MSM.882131 PMC3628141

[27] Robinson AB, Tangpricha V, Yow E, Gurion R, McComsey GA, Schanberg LE (2014) Vitamin D deficiency is common and associated with increased C-reactive protein in children and young adults with lupus: an Atherosclerosis Prevention in Pediatric Lupus Erythematosus substudy. Lupus Sci Med 1(1):e000011. 10.1136/lupus-2014-000011 25396060 10.1136/lupus-2014-000011 PMC4225734

[28] Robinson AB, Thierry-Palmer M, Gibson KL, Rabinovich CE (2012) Disease activity, proteinuria, and Vitamin D status in children with systemic lupus erythematosus and juvenile dermatomyositis. J Pediatr 160(2):297–302. 10.1016/j.jpeds.2011.08.011 21924736 10.1016/j.jpeds.2011.08.011 PMC3258326

[29] Zhang X, Guo Q, Sun S, Tang X, Shen W, Liang J et al (2024) Factors associated with 25-hydroxyVitamin D level in Chinese hospitalized patients with systemic lupus erythematosus: a retrospective cohort study. Rheumatol Int 44(10):2067–2078. 10.1007/s00296-023-05465-5 37750894 10.1007/s00296-023-05465-5

[30] Sun J, Zhang S, Liu JS, Gui M, Zhang H (2019) Expression of Vitamin D receptor in renal tissue of lupus nephritis and its association with renal injury activity. Lupus 28(3):290–294. 10.1177/0961203319826704 30691345 10.1177/0961203319826704

[31] Yu Q, Qiao Y, Liu D, Liu F, Gao C, Duan J et al (2019) Vitamin D protects podocytes from autoantibodies induced injury in lupus nephritis by reducing aberrant autophagy. Arthritis Res Ther 21(1):19. 10.1186/s13075-018-1803-9 30635032 10.1186/s13075-018-1803-9 PMC6330406

