# Supplementary material for: Vitamin D deficiency in newly diagnosed childhood-onset systemic lupus erythematosus: prevalence and clinical associations
Source: Eur J Pediatr. 2026 May 15;185(6):395. doi: 10.1007/s00431-026-07066-3 (PMC13179183; doi:10.1007/s00431-026-07066-3)
Supplement: Supplementary file 1 — Supplementary file1 (DOCX 35 KB) [file 431_2026_7066_MOESM1_ESM.docx]

**Supplemental Table 1.** Comparison of demographic characteristics, clinical features, and laboratory findings between included and excluded patients with childhood-onset systemic lupus erythematosus (N = 267)

| **Characteristic** | **Total (N = 267)** | **Included^a)^ (n = 192, 71.9%)** | **Excluded^b)^ (n = 75, 28.1%)** | ***P* value** |
| --- | --- | --- | --- | --- |
| **Demographic characteristics** |  |  |  |  |
| Age at diagnosis, y, mean (SD)^c^ | 12.7 (2.9) | 12.1 (3) | 12.1 (2.8) | 0.991 |
| Female sex | 233 (87.3) | 167 (87) | 66 (88) | 0.822 |
| BMI, kg/m^2^ | 19.1 (16.1–22.6) | 18.8 (16–21.7) | 21.3 (16.7–25.3) | **0.005** |
| **Clinical features** |  |  |  |  |
| Fever  Mucocutaneous  Hematologic  Neuropsychiatric  Musculoskeletal  Pulmonary  Cardiac  Gastrointestinal  Lupus nephritis | 149 (55.8)  199 (74.5)  213 (79.8)  21 (7.9)  74 (27.7)  31 (11.6)  34 (12.7)  34 (12.7)  132 (49.6) | 122 (63.5)  140 (72.9)  156 (81.2)  17 (8.9)  46 (24)  24 (12.5)  28 (14.6)  28 (14.6)  92 (48.2) | 27 (36)  59 (78.7)  57 (76)  4 (5.3)  28 (37.3)  7 (9.3)  6 (8)  6 (8)  40 (53.3) | **< 0.001**  0.332  0.337  0.337  **0.028**  0.468  0.147  0.147  0.448 |
| **Laboratory findings** |  |  |  |  |
| Hemoglobin, g/dL, mean (SD)^c^  WBC, ×10^3^/μL  Platelet count, ×10^3^/μL  ESR, mm/h  Creatinine, mg/dL  UPCR, mg/mg  eGFR, mL/min/1.73 m^2^  C3, mg/dL  C4, mg/dL  Anti-dsDNA antibody positivity | 9.9 (2.1)  5670 (3415–9390)  251 000 (170 250–328 000)  59 (29–81)  0.6 (0.4–0.8)  1 (0.4–4.2)  110 (80–130)  39.4 (24.7–55.2)  5.8 (3.7–8.3)  217 (81.3) | 9.6 (2.1)  5075 (3295–7742.5)  239 500 (146 000–328 750)  60.5 (33–87)  0.5 (0.4–0.8)  1.1 (0.5–4.2)  100 (80–130)  36.3 (24.1–54.2)  5.5 (3.4–8.3)  154 (80.2) | 10.8 (2)  8040 (4725–12 840)  276 000 (207 250–326 000)  47 (25.5–76.5)  0.6 (0.5–0.7)  0.9 (0.2–4.2)  100 (80–120)  44 (32.4–58.4)  6.4 (4.4–9.3)  63 (84) | **< 0.001**  **< 0.001**  0.05  0.063  0.12  0.495  0.186  0.101  0.182  0.475 |
| **Disease activity at diagnosis** |  |  |  |  |
| SLEDAI-2K | 12 (8–17) | 13 (8–17) | 12 (8.5–16) | 0.5 |

^a)^ Patients with c-SLE who had a serum 25-OHD level obtained at the time of diagnosis and were not receiving vitamin D supplementation.
^b)^ Patients excluded because a serum 25-OHD level was not obtained at the time of diagnosis (*n* = 71) or because of concurrent vitamin D supplementation (*n* = 4).
Data are presented as *n* (%) for categorical variables, mean (SD) for normally distributed continuous variables^c)^, or median (IQR) for nonnormally distributed continuous variables.
Comparisons between the included and excluded groups were performed using the independent samples *t* test or Mann–Whitney *U* test for continuous variables and the chi-square test for categorical variables.
Bold *P* values indicate statistical significance (*P* < 0.05).
^c)^ Age at diagnosis and hemoglobin are the only variables reported as mean (SD); all other continuous variables are reported as median (IQR).
**Abbreviations:** 25-OHD, 25-hydroxyvitamin D; anti-dsDNA, anti–double-stranded DNA; BMI, body mass index; C3, complement 3; C4, complement 4; c-SLE, childhood-onset systemic lupus erythematosus; eGFR, estimated glomerular filtration rate; ESR, erythrocyte sedimentation rate; IQR, interquartile range; SD, standard deviation; SLEDAI-2K, Systemic Lupus Erythematosus Disease Activity Index 2000; UPCR, urine protein-to-creatinine ratio; WBC, white blood cell count

**Supplemental Figure 1.** Flow Diagram of Patient Selection and Study Cohort Formation

c-SLE patients assessed for eligibility

N=267

Vitamin D nondeficient

N=66 (34.4%)

Excluded

- No baseline serum 25-OHD level, N=71
- Receiving vitamin D supplementation, N=4

Included in final analysis

N=192

Vitamin D deficient

N=126 (65.6%)
